# Highlight on Dr. Rhonda Voskuhl: a pioneer in the exploration of sex differences in the immune response

**DOI:** 10.1186/s13293-026-00910-z

**Published:** 2026-05-08

**Authors:** Shannon E. Dunn

**Affiliations:** 1 Department of Immunology, the University of Toronto, Toronto, ON Canada; 2 Biological Platform, Sunnybrook Research Institute, Toronto, ON Canada; 3 Women’s College Research Institute, Toronto, ON Canada

Dr. Rhonda Voskuhl is a neuroimmunologist and a neurologist at the University of California, Los Angeles (UCLA). It has been almost three decades since Dr. Voskuhl published her first paper on the sex differences in an animal model of multiple sclerosis (MS) called experimental autoimmune encephalomyelitis (EAE) [1]. Dr. Voskuhl has received a number of awards and accolades for her discoveries of anti-inflammatory and neuroprotective effects of gonadal hormones and the role of the sex chromosomes in regulating sex differences in EAE as well as the translation of these research findings into MS clinical trials. This commentary serves to celebrate how her research into autoimmune mechanisms in a simple inbred mouse strain called Swiss Jackson Lambert (SJL) helped define the key ways in which T cell adaptive immune responses differ between a male and female.

As a medical student, Dr. Voskuhl was fascinated by the brain as well as why the immune system would attack itself, so when she became a Resident, Neurology with a specialty in MS was a natural fit. During this time at the University of Texas Southwestern, she took part in a research rotation and became hooked on research. Through this research, she became interested in how autoreactive T cells get activated in some people and not in others. After completing her residency, she pursued a post-doctoral fellowship in immunology in MS at the National Institutes of Health (NIH) under the mentorship of Dr. Henry McFarland. At the time, the interest was (and still is) to understand the nature of the autoimmune reaction in MS. Following the discovery that T cell clones reactive against the major myelin antigen, myelin basic protein (MBP), could induce an-MS like disease in mice [2], Dr. Voskuhl set out to characterize the T cells specific to MBP and a variant of this protein that contains exon 2 (called X2 MBP) that is expressed only during development and remyelination [3–6]. The idea is that potentially, the immune system would be less tolerant against this variant of MBP because it is not expressed in the brain at steady state. This research involved the painstaking isolation of MBP-specific T cell clones in those affected and not affected by MS in multiple-case families and in unrelated individuals, and sequencing the T cell receptors. The results of some of this early research confirmed that MBP-reactive T cells had a T helper 1 (Th1)-like profile similar to the MBP-reactive T cells described to induce EAE in mice [4] and that vaccination of mice of the SJL mouse strain with the X2 MBP variant, like MBP could cause MS-like disease [7] ; however, MBP- (and X2 MBP)-specific T cells were puzzlingly just as abundant in healthy people as they were in the patients with MS [6]. Thirty years later, researchers are still trying to figure out whether MBP is a relevant autoantigen in MS. Recent papers suggest that MBP-specific T cells may be activated in response to recognition of peptides encoded by Epstein-Barr virus (EBV) via a mechanism of molecular mimicry [8,9].

During the interview with Dr. Voskuhl, I asked her whether she had any advice that she could share to young fellows or PhD students starting in their research careers. She said, “the best thing you can do is take a walk by yourself in the woods and think about what the important outstanding questions are in your field, that, if solved, will have an impact on the quality of life of patients.” She continued, “You don’t want to follow the herd and study what everyone else is studying, because then your research will only be incremental.”

She applied this approach to her own studies. During her fellowship, clinical trials were being run for MS, and these trials recruited women and men with a 60:40 or 70:30 split. This led her to ask the simple question, “Why do females get more MS compared to males?“. Her training in human immunology at the NIH provided an important perspective on just how heterogeneous the T cell compartment is in a human. She reasoned that it would likely be easier to study the effect of sex on central nervous system (CNS) autoimmunity in an inbred mouse strain that has identical autosomes and is housed under controlled environmental conditions. The SJL model was a natural choice since it is an inflammatory strain that is very susceptible to EAE when immunized with MBP. At the time, there was general knowledge among those working with the model that females were more susceptible to EAE. For this reason, the convention at the time (and still is) that most MS researchers used female rodents for their studies. Similarly, there were also clues from the literature that T cells from female rodents were more easily sensitized to alloantigens during transplant as well as upon vaccination with model antigens such as ovalbumin [10, 11]. Dr. Voskuhl decided to investigate whether there were also sex differences in the central nervous system (CNS) inflammatory response that occurs downstream of myelin-specific T cell sensitization in EAE. For these studies, she expanded MBP-specific T cells in donor SJL mice (either male or female) by vaccinating them with MBP and a strong adjuvant and then collected the draining lymph node cells, re-exposed the T cells to MBP, and then transferred these cells into male or female SJL recipient mice. She observed that female SJL recipient mice developed more severe clinical and histological EAE disease compared to the males [1]. She also presented evidence that SJL mice are particularly well-suited for T cell transplantation studies, since female SJL mice do not reject male cells, which can occur when such experiments are done in other mouse strains [1].

As is often the case in science, independent groups ask the same research questions, and findings converge. Just prior to Dr. Voskuhl’s article on sex differences in adoptive transfer EAE was published, Dr. Stephen Stohlman at the University of Southern California and his then PhD student Daniel Cua (who later went on to fame for discovering the IL-23-Th17 axis in autoimmune inflammation) [12] reported that young adult male SJL mice are resistant to sensitization with myelin antigens as compared to female SJL mice [13]. This protection in males could be transferred to healthy and correlated with a greater secretion of cytokines associated with anti-inflammatory T helper 2 cells (IL-10 and IL-4) and lowered expression of the T helper 1 cytokine, IFN-γ, by the draining lymph nodes [13, 14]. At around the same time, Dr. Halina Offner and her trainee Bruce Bebo at the University of Oregon published that male SJL mice were less likely than females to experience relapses during EAE; which was similarly associated with greater mRNA expression of IL-4, in the male lymph nodes [15]. It is important to remember that at the time of these discoveries, IL-10, which is now recognized to be one of the most important cytokines involved in tolerance and prevention of autoimmunity, was thought to be a cytokine associated with the Th2 cell lineage. Furthermore, FoxP3 T regulatory cells, which are essential to preventing autoimmunity and produce IL-10, had yet to be discovered [16]. More recent investigations have further refined these observations in the SJL mouse by showing that there are two different biologies that are more dominant in males. First, male SJL mice develop enhanced Th2 immunity due to greater activity of type 2 innate lymphoid cells, which are fast-acting cells in the innate immune system that can produce copious amounts of IL-4 [17]. In addition, males have a greater frequency of FoxP3^+^ T regulatory T cells, which express greater levels of IL-10 and the co-inhibitory molecule CTLA-4 [18].

In 1996, Dr. Voskuhl was recruited to UCLA and continued her work on sex differences and investigated mechanisms underlying the sex differences in the sensitization of myelin-specific T cells in SJL mice in EAE. She observed that T cells expanded from draining lymph nodes of female myelin-sensitized mice were more able to transfer EAE compared to male cells [19]; an observation also made by the Offner group [20]. Closer examination of the phenotype of the MBP-sensitized lymph node cells revealed a number of T cell features that differed between the males and females. First, the proliferative responses of the T cells upon being re-exposed to MBP were greater in the female. This meant that either the MBP-specific T cell pool is larger in a female or that the individual T cells have a greater capacity to proliferate. In addition, similar to the previous reports of the Stohlman group, the draining lymph node cells from the females produced greater levels of the pro-Th1 and Th17 factor IL-12p40 and the Th1 cytokine IFN-γ upon re-stimulation with MBP. Others found that females also exhibited a Th1 bias in the immune response in MS [21]. Altogether, these findings by the Voskuhl, Stolhman, and Offner groups gave birth to the notion that the female immune system is biased towards Th1 immunity, whereas the male immune system is biased towards a non-pathogenic Th2 response. These findings therefore provided an understanding of why females are more poised to fight infection (where a Th1 response is more helpful) and, at the same time, are more prone to autoimmunity (where Th1 responses can be pro-inflammatory).

Next, Dr. Voskuhl set out to dissect the mechanisms underlying these sex differences in immunity in EAE. “When you are trying to dissect a sex difference phenotype, it is relatively simple: it is either driven by differences in gonadal hormones or sex chromosomes,” says Dr. Voskuhl. “Examining the influence of gonadal hormones is easier since you can remove the gonads in mice to reduce hormone levels and then can add-back individual hormones to see which one is driving your phenotype”. Her lab group started to conduct such experiments in the EAE model in SJL mice. She found that castration of the male SJL mice worsened EAE [22], whereas treatment with the androgen receptor agonist dihydrotestosterone (DHT) attenuated EAE [23]; this suggested that the greater androgen levels in males protects against autoimmunity. She saw similar protective effects of DHT in females that correlated with greater production of the anti-inflammatory cytokine IL-10 by the transferred Th1 cells, as well as lowered production of the pro-Th1/Th17 cytokine IL-12p40. If we examined these data through today’s lens, we would likely recognize this to be an enhanced T regulatory response in DHT-treated mice.

In conducting the hormone add-back experiments in the female ovariectomized mice, Dr. Voskuhl was using estriol instead of estradiol, which is the more commonly-studied estrogen that fluctuates with the menstrual cycle. I asked her why she studied the effects of estriol. She responded:

“Even when I am planning basic experiments, I always have the patient at the forefront in mind—so I wanted to test an estrogen that was safe and therefore had the potential to become a therapy in the future. At the same time, I was thinking about another outstanding question in the field of MS; Why do MS symptoms improve in women during late pregnancy? Studies by Confavreux and colleagues [24] showed very strong evidence that relapse rates in women with MS decline in the third trimester of pregnancy. Estriol is a form of estrogen made by the fetal placental unit that rises throughout pregnancy and is present at the highest levels in the third trimester. It is also a weak estrogen and doesn’t hit the estrogen-receptor alpha as strongly as beta-estradiol; and instead binds estrogen receptor-beta. This was important since estrogen receptor-alpha is expressed in breast tissue can promote breast cancer in women.”

Dr. Voskuhl’s group showed that, when provided with pregnancy levels of estriol, female mice were protected from EAE and that this was associated with greater production of the anti-inflammatory cytokine IL-10 [25]. These findings that both androgens and estriol were anti-inflammatory also meant that both types of gonadal steroids had the potential to be therapeutic avenues for MS.

What is truly remarkable about Dr. Voskuhl’s early research is how rapidly she translated these basic research findings of gonadal steroids in EAE into human clinical trials. Soon after describing anti-inflammatory effects of androgens and estriol in EAE, Dr. Voskuhl conducted small pilot studies of these hormones in MS patients. In these trials, each person served as its own control, and measurements were done at baseline and after starting treatment. Beyond recognizing important neuroprotective effects of steroids, the trials provided the first glimpse of the effects of androgen (and estriol) therapy on the immune system in humans [26–28]. For example, similar to findings in SJL mice [14], androgen therapy attenuated the delayed-type hypersensitivity (Th1-type) responses [26,28]. In this same trial, her group observed that androgen therapy caused a decline in CD4^+^ T cells, which are the T cells that initiate immune responses, and increased natural killer (NK) cells, a type of innate immune cell that is specialized in lysing tumour or viral infected cells. The finding that females have greater T helper (or CD4^+^) T cells and lower levels of NK cells in blood has been recognized to be a sex-dependent immune trait in humans [29, 30].

Though this early work certainly pointed to a dominant role for gonadal steroids in mediating the sex differences in immunity, this work did not preclude a role for sex-chromosome-encoded genes. As Dr. Voskuhl explained:

“Dissecting the effect of sex chromosomes on immunity is trickier. If you remove the gonads from an adult or pre-pubescent mouse and still see a sex difference in your biological process, it doesn’t mean that you are studying a sex chromosome effect. This is because the organism is also exposed to sex steroids during early stages of development.”

So, to study the effect of sex chromosomes as a single variable, she had to control for differences in hormones not only in adulthood but also during development. This was made possible when Dr. Voskuhl met Dr. Arthur Arnold (also at UCLA) and learned about the four-core genotype model (FCG) that he was working with, she saw an opportunity to use this model to untangle the effects of gonadal hormones and sex chromosomes on immunophenotypes [31]. In the FCG mouse, a spontaneous mutation has occurred where the testes-determining gene Sry was lost from the Y chromosome and inserted on an autosome. When males having this genotype are mated with XX females, the following combinations of mice are generated: XX mice that have ovaries, XY-Sry^-/-^ mice that develop ovaries, XX-Sry mice that develop testes, and XY Sry^-/-^ mice that express Sry and develop testes. “With this model, mice are living with these sex chromosome or gonadal patterns throughout development, and you can discretely separate a hormone effect from a chromosome effect” says Dr. Voskuhl. She bred these mice onto the SJL background, immunized them with her favorite antigen, MBP, and then examined the recall proliferative and cytokine responses of the draining lymph node cells to MBP ex vivo. These studies validated her previous observations that mice with ovaries (XX or, XY Sry^-/-^) exhibit greater T cell and Th1 cytokine production compared to the mice that have testes (XX-Sry or XY Sry^-/+^)^31^. This was found to be largely due to dominant, immunosuppressive effects of androgens acting in a male, which mask any influences of the underlying sex chromosome complement in the gonad intact mice [31]. When the influence of gonadal hormones was removed by gonadectomizing the FCG mice, the effects of sex chromosomes on immunity were revealed [31]. This report by Voskuhl and Arnold represents the first dissection of the role of sex chromosome complement on the immune system in mice using the FCG mouse model. The FCG model has since become a go-to model in the field to dissect sex chromosome from gonadal hormone effects. Notably, some controversy recently emerged about the use of FCG strain after it was discovered that a translocation event had occurred in a line maintained on the C57BL6/J background at Jackson Laboratory: a section of chromosome X containing 7 genes was duplicated and inserted itself in the Y chromosome [32]. Since Dr. Voskuhl’s studies were done in the SJL version of this mouse, it would not alter the conclusions of her research [33]. Laudably, her colleague, Dr. Art Arnold has worked with other researchers to ensure that a version of FCG mouse on the C57BL6/J background without this translocation is readily available to investigators at the Jackson laboratory.

Drs. Voskuhl and Arnold proceeded to use the FCG mice on the SJL background to study the role of the sex chromosomes (with the influence of gonadal hormones removed) on EAE and in pristane-induced systemic lupus erythematosus (SLE) in SJL mice. They found that mice with an XX chromosome complement presented with more severe EAE compared to those having an XY, due to the increased sensitization of the myelin-specific T cells in the XX mice. Interestingly, the immunostimulatory effect of XX was not observed when FCG mice were crossed on the C57BL6/J strain, which does not exhibit a sex difference in EAE development [34]. In addition, the XX SJL female mice succumbed to pristane-induced lupus at a faster rate with greater levels of dsDNA antibodies compared to mice having an XY complement [35]. Related was their findings in another autoimmune strain, the NZM2328, where XX compared to XY mice had more severe spontaneous lupus [36]. This recognition of a stimulatory role for the X chromosome, together with human investigations at the time that described an increase in lupus susceptibility in Klinefelter’s men (those having an XXY genotype instead of XY), put the spotlight on the X chromosome and X-encoded genes as potential contributors to the heightened immune response in females [37]. When asked about what was special about the SJL mouse, Voskuhl said, “the pro-inflammatory effect of having two XX chromosomes is more easily seen in this strain, and therefore it is a better model of the pro-inflammatory characteristics of the immune system that are seen in a female human.”

Next the search was on to identify the “immunostimulatory” genes on the X chromosome. Dr. Voskuhl recounted that she had read a paper that described the genes that are differently expressed between male and female cells, and what struck her was that there were a lot of them. Not all of them had hormone-sensitive gene regulatory regions. When she looked for genes that are differentially expressed between the sexes on the sex chromosomes, there were only a handful that escaped the process of X inactivation—one of these genes was *Kdm6a* (or *KDM6A* in humans). “Knowing that the XX chromosome complement is immunostimulatory, the histone demethylase *Kdm6a* was a good candidate, since it is a histone-modifying enzyme, and therefore can broadly regulate autosomal gene expression." Dr. Voskuhl proceeded to knockout *Kdm6a* first in T cells, and this attenuated T cell immunity and the development of EAE in female mice [38]. More recently, she reported on the effect of deletion of *Kdm6a* specifically in microglia [39], which are brain immune cells that play a role in inciting neuroinflammation. Her group observed that deletion of this gene in microglia reduced microglia expression of disease-associated markers and ameliorated brain pathology, and this occurred specifically in females. This suggested that *Kdm6a* promotes inflammation in females. Furthermore, having the patient in mind, she also tested a known, safe therapy, Metformin, which, amongst its activities, inhibits Kdm6a activity. She found that metformin inhibited microglia activation only in the female mice. Voskuhl added to this by saying:

“We now think that greater Kdm6a expression is the X factor that is contributing to greater inflammation in the female. We think that estrogens act to temper this pro-inflammatory effect until females reach menopause. At menopause and thereafter, there is neuroinflammation involving microglia and astrocyte activation with synaptic dysfunction, potentially explaining why 60–70% of otherwise healthy menopausal women have cognitive symptoms after menopause.”

In parallel, work by others has recognized other X-encoded genes that are important in other autoimmune diseases. For example, the RNA sensor toll-like receptor 7 (TLR7) is expressed at greater levels in female B cells and plasmacytoid dendritic cells and promotes SLE through increased type I IFN production [40, 41]. Dr. Voskuhl’s group has also provided a molecular mechanism for the paradoxical higher expression of certain X-encoded genes (e.g. FoxP3) in a male [42, 43]. They reported that the paternally-inherited X chromosome (Xp) exhibits greater methylation than the maternally-inherited X (Xm). Males, who only inherit Xm, have overall less methylation at certain gene loci compared to females that randomly inactivate Xp or Xm [42, 43].

Altogether, the work of Dr. Voskuhl and her colleagues, who used a simple inbred mouse strain to study sex differences in EAE, has described the key components in T cell adaptive immunity that differ between males and females. What is extraordinary about this work is that many of the sex differences defined in this “pro-inflammatory” strain of the SJL mouse were later described to be true in the human immune system. For example, the finding that the size of the autoreactive T cell pool is larger in a female than a male mouse [19] has been described for healthy humans [44]. The finding of a greater frequency of Th1-specific cells in females with EAE was also observed in human studies that characterized viral-specific [45] or autoreactive T cell responses [21] in blood. The finding that the individual female CD4^+^ T cells were more prone to secrete IFN−γ and other effector molecules was also later shown to be true for T cells in humans [46, 47], and it has since been recognized to be a property that is better retained by female murine CD8^+^ T cells in the tumor microenvironment [48, 49]. The finding that male SJL T cells produce greater IL-10 due to androgens also preceded the discovery that men have more Treg within the CD4^+^ T cell compartment [50, 51] and are more prone to differentiate towards type I regulatory cells [52].

When asked about her future research goals, Dr. Voskuhl is currently planning advanced human clinical trials to test estriol in people. Though the anti-inflammatory effects of this hormone were some of her original findings, in other work, her group also described potent neuroprotective effects of estriol and estrogen-receptor-beta agonists. She added:

“I have always used the patient (bedside) to bench to bedside approach– what I choose to study always starts with the patient. We had learned from our early studies using estriol, that in addition to having anti-inflammatory effects, this hormone was sparing neurons by calming inflammation in the brain and was providing an environment that was supporting remyelination. We knew that estriol preferentially hits ER-beta. We tested specific ligands for ER-alpha and ER-beta and mapped out the action of estrogens and found that both ER-alpha and ER-beta had neuroprotective effects in pre-clinical models (see refs [53–57]). So we started with the patient need (the requirement of a safe treatment that would be both anti-inflammatory and neuroprotective), we did the benchwork, and mapped out the effects of the different hormones on different cell types in the animal models, and now are at the bedside stage, where we are designing therapies to treat MS patients with estriol. We are particularly interested in treating women during perimenopause and thereafter, since the progressive stage of this disease tends to start around age 50, and menopausal women will be without ovarian hormones starting at mean age 51.5 and for the rest of their life.”

In sum, although the reason females are more prone to autoimmunity is often described as a mystery, we actually have a very good understanding of why this is–largely because of this pioneering work done in mice and humans by Dr. Voskuhl and her colleagues. At the same time, her basic research of gonadal hormones in animal models has created new hope for those suffering from MS and other neurodegenerative conditions.
